# Supplementary material for: Structural and biochemical characterization establishes a detailed understanding of KEAP1-CUL3 complex assembly
Source: Free Radic Biol Med. 2023 Aug 1;204:215–25. doi: 10.1016/j.freeradbiomed.2023.04.021 (PMC10564622; doi:10.1016/j.freeradbiomed.2023.04.021)
Supplement: Multimedia component 1 [file mmc1.pdf]

Appendix A. Supplementary data

Structural and biochemical characterization establishes a detailed understanding of KEAP1-CUL3 complex assembly

Table S1. Diffraction data collection and refinement statistics

Table S2. Equilibrium dissociation constants (K_D values) determined in KLHL11$_{BTB-BACK}$ / CUL3$_{NTD}$ TR-FRET protein displacement assay.

Table S3. Equilibrium dissociation constants (K_D values) determined in KEAP1$_{FL}$ / CUL3$_{NTD}$ TR-FRET protein displacement assay.

Fig. S1. Crystal packing may create steric hindrance for the CUL3 N-terminal extension.

Fig. S2. Biolayer interferometry (BLI) experiments with certain KEAP1 and CUL3 constructs showed significant deviation from a standard Langmuirian 1:1 model.

Fig. S3. KEAP1$_{FL}$ dimerization assay.

Table S1. Diffraction data collection and refinement statistics

	KEAP1-CUL3
**Data collection[a]**	
Beamline	Diamond I03
Wavelength (Å)	0.9763
Spacegroup	C2 2 21
Cell dimensions	
a/b/c (Å)	41/233.38/164.71
α/β/γ (°)	90/90/90
Resolution range (Å)	40.38 - 3.45 (3.573-3.45)
Total reflections	70080 (6938)
Unique reflections	10902 (1049)
Multiplicity	6.4 (6.6)
Completeness (%)	99.55 (99.24)
Mean I/σI	12.96 (1.46)
R_{merge}	0.08226 (1.522)
R_{meas}	0.08983 (1.653)
R_{pim}	0.03557 (0.6393)
CC1/2	0.999 (0.611)
**Refinement[a]**	
Reflections used in refinement	10899 (1049)
Reflections used for R_{free}	520 (60)
MR model	4AP2
Copies in ASU	1
R_{work}/R_{free} (%)	23.8/28.8 (33.8/36.2)
CC work/CC free	0.971/0.950 (0.788/0.526)
Number of non-hydrogen atoms	3882
Protein residues	502
rmsd	
Bond lengths (Å)	0.011
Bond angles (°)	1.44
Ramachandran favoured (%)	92.14
Ramachandran allowed (%)	7.06
Ramachandran outliers (%)	0.81
Rotamer outliers (%)	7.87
Clashscore	8.57
Average B-factor ($Å^2$)	167.14
Protein Data Bank code	5NLB

[a] Values in parentheses are for the highest resolution shell.

Table S2. Equilibrium dissociation constants (K_D values) determined in KLHL11$_{BTB\text{-}BACK}$ / CUL3$_{NTD}$ TR-FRET protein displacement assay.

Protein construct	K_D (nM)	K_D 95% CI (nM)
KLHL11$_{BTB\text{-}BACK}$	14	13-15
CUL3$_{NTD}$	8	7-9
CUL3$_{NTD\Delta22}$	1,840	1,702-1,990
KEAP1$_{BTB\text{-}3\text{-}box}$	720	650-797
KEAP1$_{BTB\text{-}BACK\text{-}Kelch}$	798	730-872

Table S3. Equilibrium dissociation constants (K_D values) determined in $KEAP1_{FL}$ / $CUL3_{NTD}$ TR-FRET protein displacement assay.

Protein construct	K_D (nM)	K_D 95% CI (nM)
$CUL3_{NTD}$	129	117-142
$CUL3_{NTD\Delta22}$	13,783	12,047-16,119
$KEAP1_{BTB\text{-}3\text{-}box}$	1,042	943-1,151
$KEAP1_{BTB\text{-}BACK\text{-}Kelch}$	396	365-430

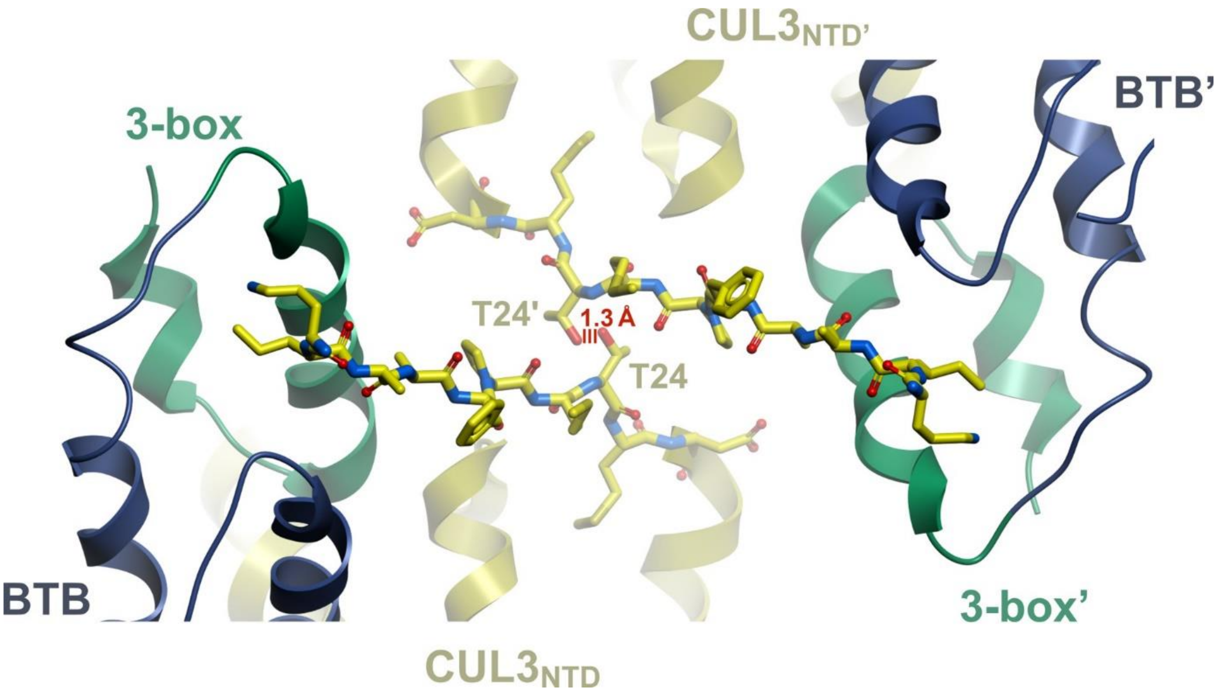

Fig. S1. Crystal packing may create steric hindrance for the CUL3 N-terminal extension. Two KEAP1-CUL3 complexes are shown in the crystal lattice. The $CUL3_{NTD}$ from PDB 4AP2 was modelled onto the $CUL3_{NTD}$ subunits of the KEAP1 complex to reveal the expected position of the CUL3 N-terminal extension if bound to the 3-box groove of KEAP1. This model reveals a potential clash between the N-terminal extensions in two adjacent $CUL3_{NTD}$ chains (distance 1.33 Å). Any steric hindrance could have promoted the apparent disorder in this CUL3 region.

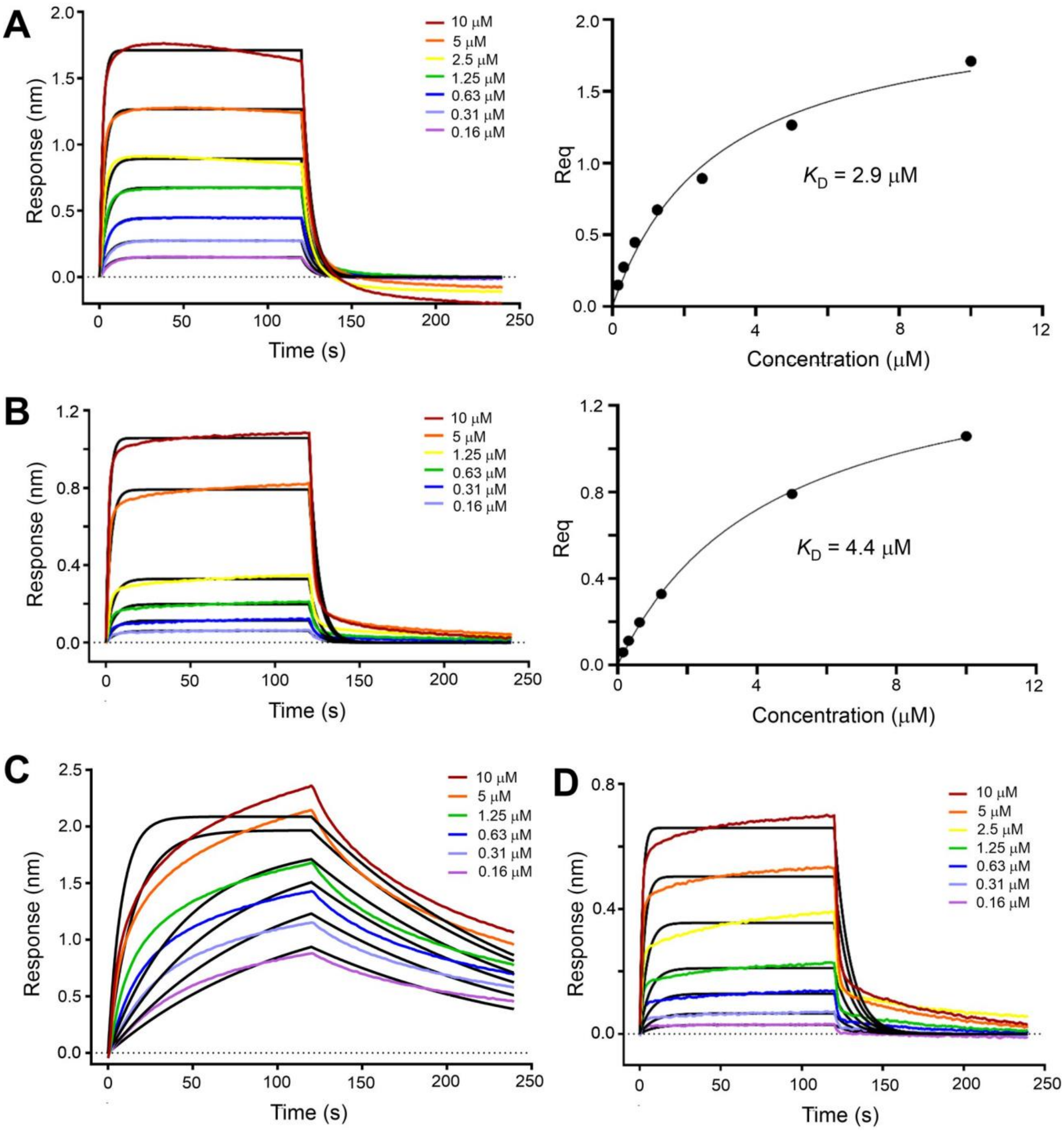

Fig. S2. Biolayer interferometry (BLI) experiments with certain KEAP1 and CUL3 constructs showed significant deviation from a standard Langmuirian 1:1 model. Binding measurements were recorded on an Octet RED384 instrument (FortéBio). (A) Biotinylated $KEAP1_{BTB\text{-}3\text{-}box}$ was immobilized and the binding of $CUL3_{NTD}$ showed an apparent steady state K_D = 2.9 μM (95% CI 1.7-5.1 μM) and binding kinetics of k_{on} = 3.66 x 10^4 $M^{-1}s^{-1}$, k_{off} = 1.89 x 10^{-1}

s^{-1}, ${}^{App}K_D$ = 5.2 μM. (B) Biotinylated $KEAP1_{BTB\text{-}3\text{-}box}$ was immobilized and the binding of full length CUL3-RBX1 complex showed an apparent steady state K_D = 4.4 μM (95% CI 3.7-5.3 μM) and binding kinetics of k_{on} = 3.32 x 10^4 $M^{-1}s^{-1}$, k_{off} = 1.87 x 10^{-1} s^{-1}, ${}^{App}K_D$ = 5.6 μM. (C) Biotinylated $CUL3_{NTD}$ was immobilized on a streptavidin-functionalized sensor tip and binding to serial dilutions of $KEAP1_{BTB\text{-}BACK\text{-}KELCH}$ (residues 48-624) was assessed. Significant deviation from a standard Langmuirian 1:1 model was observed preventing reliable K_D determination. (D) Biotinylated $KEAP1_{BTB\text{-}3\text{-}box}$ was immobilized and the binding of $CUL3_{NTD\Delta 22}$ was assessed. Significant deviation from a standard Langmuirian 1:1 model was observed preventing reliable K_D determination.

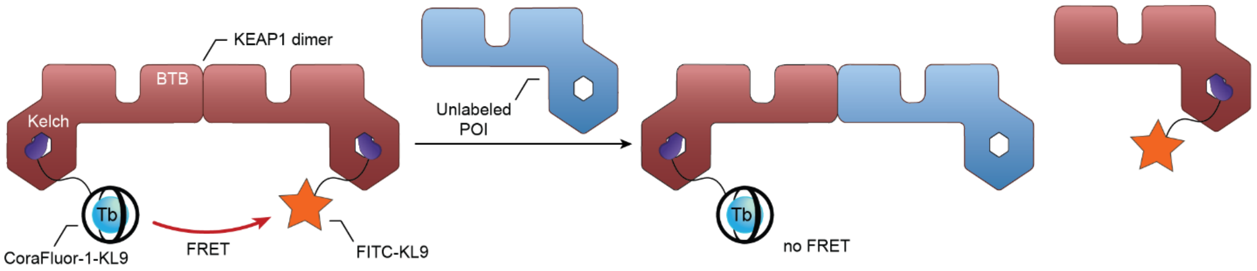

Fig. S3. KEAP1$_{FL}$ dimerization assay. Schematic representation of the KEAP1 dimerization assay employing KEAP1$_{FL}$ and a mixture of CoraFluor-1- and FITC-labeled NRF2-derived peptides (LDEETGEFL-CONH$_2$; CoraFluor-1-KL9 and FITC-KL9, respectively). In this assay, KI-696, a potent KEAP1-Kelch domain inhibitor, acts as a positive control to disrupt TR-FRET signal (see Fig. 7A).
